# Is a driver’s license age waiver worth a teen’s life?

**DOI:** 10.1186/s40621-018-0146-y

**Published:** 2018-04-10

**Authors:** Dawn M. Porter, Beverly K. Miller, Samantha H. Mullins, Mary E. Porter, Mary E. Aitken

**Affiliations:** 1 Center for Applied Research and Evaluation, Department of Pediatrics, University of Arkansas for Medical Sciences, Little Rock, AR 72202 USA; 2 Injury Prevention Center, Arkansas Children’s, Little Rock, AR 72202 USA; 3 Arkansas Children’s Research Institute, Little Rock, AR 72202 USA

**Keywords:** Teen, Age waiver, Graduated driver license

## Abstract

**Background:**

Motor vehicle crashes are the leading cause of death for teens 14–19 years of age, with younger teen drivers at higher risk than older teens. Graduated driver licensing has been proven to reduce teen driver-related motor vehicle crashes and fatalities. Arkansas allows parents to request age waivers, which allow a teen to obtain a license for independent driving before the sixteenth birthday. The objectives of this study were to: (1) determine the prevalence of age waivers issued in Arkansas and (2) determine motor vehicle crash risks associated with 14 and 15 year old drivers.

**Methods:**

This is a brief report on an informative query exploring risk factors related to age waivers. Publicly available databases were utilized for across state comparisons. The Web-based Injury Statistics Query and Reporting Systems (WISQARS) was utilized to calculate motor vehicle crash crude death rates. National Highway Traffic Safety Administration data were utilized to identify seat belt use rates. The Fatal Analysis Reporting System (FARS) was utilized to identify crash fatality risks for 14 and 15 year old drivers in Arkansas (*N* = 24). Age waiver data were obtained from the Arkansas Driver Control Administration. De-identified data on fatal crashes and rates of age waiver issuance in Arkansas for 14 and 15 year olds from 2004 through 2016 were calculated.

**Results:**

We reviewed crash data for 14 and 15 year old drivers in Arkansas between 2004 and 2014 to determine fatality risks. Thirty-one out of seventy-five counties in Arkansas were above the state age waiver issuance rate of 30.4 per 1000 14 to 15 year old teens. Among the four states that had similar age waivers for 14 to 15 year olds, Arkansas had the highest motor vehicle death rate of 10.2 per 100,000 young teens and the lowest seat belt use rate at 73%.

**Conclusions:**

Arkansas had the highest reported teen crash fatality rates among 4 states with age waivers. The volume of age waivers issued in Arkansas is concerning. Further research is needed to understand parental motivation when asking for age waivers and their level of awareness of the risks involved.

## Background

Motor vehicle crashes are the leading cause of death for youth ages 14–19 years old (Centers for Disease Control and Prevention, 2009a). Inexperience, distractions, and immaturity contribute to motor vehicle crash risks for teens (Ferguson, 2003; Lin & Fearn, 2003; Simons-Morton, 2002; Sturrs et al., 2001; Williams, 2003). Younger drivers are at much higher risk, with 16 year old drivers having crash rates that are 3 times higher than 17 year old drivers and 5 times higher than 18 year old drivers (Mayhew et al., 2003). To address these risks, all 50 states and the District of Columbia have enacted three-stage graduated driver licensing (GDL) laws (Insurance Institute for Highway Safety, 2017). Although provisions vary among states, the laws are intended to restrict a newly licensed teen driver’s exposure to the riskiest driving conditions while increasing the supervised driving period (Preusser & Tison, 2007). Most common components include limits on nighttime driving, restrictions on passengers, and supervised driving periods (Ferguson et al., 2007). GDL has been found to reduce motor vehicle crashes by 19% (Salam et al., 2016), and especially impacts crashes associated with the risks restricted as components of GDL (McCartt & Teoh, 2015). GDL programs with stronger restrictions are associated with the greatest fatality reduction (Russell et al., 2011). Parental supervision of young drivers during the learning period is a strong factor in reducing teen driving-related crashes. Young drivers with fewer hours of supervised driving time have more crashes than those with higher amounts (Gulliver et al., 2013). Parents and teens who do not consider driving a risk to teens’ safety or health are less likely to have significant parental involvement and limit-setting, and may demonstrate a resistance to policies that support supervision (Laird, 2014).

The Arkansas GDL was implemented in 2009. Arkansas teens as young as 14 years can get a learner’s license, which requires the teen driver to have an undefined amount of supervision for six months by an adult 21 years of age or older in the front seat of the vehicle at all times (Arkansas Act 394 of 2009, 2009). Despite the restrictions placed on young drivers by the GDL and its proven effectiveness, an age waiver can be issued to teens in Arkansas as young as 14 years old. Age waivers allow a teen to drive without adult supervision for specified purposes and to designated locations such as school, work, or medical facilities. In essence, the teen is allowed to both obtain a driver license two years earlier than his or her peers and by-pass the 6-month supervised driving restriction of GDL.

To obtain an age waiver, the parent and teen must complete several requirements. The teen must already have a valid learner license for at least 6 months with no citations. The parent must complete an application including written documentation of the need for the age waiver from a verifying body. For example, if the age waiver is for the teen to attend medical appointments a physician must provide verification of need. Additionally a written document must be obtained from someone outside the family who knows the teen, knows of the need of the waiver, and can endorse the teen as a responsible driver. After gathering all documents, parents must request and attend a meeting with a Driver Control Hearing Officer (DCHO) at the Arkansas Driver Control Administration who reviews the documents and either approves or denies the age waiver request.

An age waiver allows a teen to drive without a licensed adult in present. The teen is required to carry age waiver documentation with their driver license at all times. The age waiver has clearly defined stipulations as to the location to which a teen may drive. If the teen receives a citation or has a crash involving injury to self or others, the age waiver is revoked with no opportunity to apply for another waiver.

We identified no previous studies that have been conducted to determine the extent of issued age waivers to young drivers or to determine influencing factors for parents in requesting age waivers. Arkansas DCHO has indicated that some Arkansas parents are seeking age waivers for convenience, and that parents are not well-informed of the increased risks. The objectives of this project were: (1) determine the prevalence of age waivers issued in Arkansas and (2) identify patterns of motor vehicle crash risks associated with 14 and 15 year old drivers.

## Methods

### Data analysis

Retrospective methods were utilized as formative evaluation to better understand the risks associated with driver’s ages 14–15 years. Because the work was considered program evaluation and only publically available aggregated data were utilized, IRB approval was not required. Data from 2006 to 2015, provided by Arkansas Driver Control Administration, determined state and county rates for issuance of age waivers (Arkansas Integrated Revenue Systems, 2016). Age and county of issuance were the main data points collected. We utilized the US Census data by 14 and 15 year olds for Arkansas, by county, using the standardized rate calculations (United States Census Bureau, n.d.). Because these are state held data sets there are no national databases available to access other states age waiver issuance. We utilized the Web-based Injury Statistics Query and Reporting Systems (WISQARS), a Centers for Disease Control and Prevention (CDC) database), to calculate motor vehicle crash death crude rates among 14 and 15 year olds, between Arkansas, Texas, Maine and Tennessee, as these four states had comparable age waiver provisions (Centers for Disease Control and Prevention, 2009a). We also utilized the National Highway Traffic Safety Administration (NHTSA) data to report seat belt use rates among all ages of drivers and passengers, between all four states. Seatbelt use rates among teens are not available as a separate data set from NHTSA (Pickrell, 2017). Arkansas fatal crash risks percentages were calculated among 14 and 15 year old drivers for the period 2004 and 2014, by utilizing the Fatal Analysis Reporting System (FARS), a NHTSA data base (Fatal Analysis Reporting Systems, 2009). The variables obtained to assess “fatality risk” were: time of day, seat belt use at time of crash, ejection from vehicle, sex and gender of driver, and alcohol involvement when reported.

## Results

### Age waivers in Arkansas

**Fig. 1:**
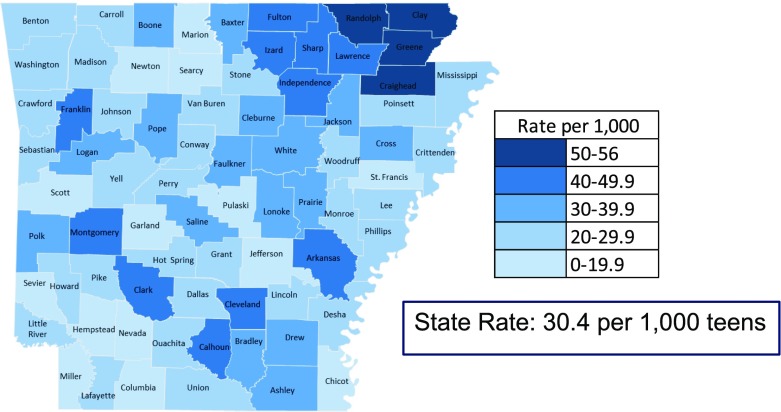
Age Waiver Rates for Drivers Ages 14–15, Arkansas, 2006–2015

**Fig. 2:**
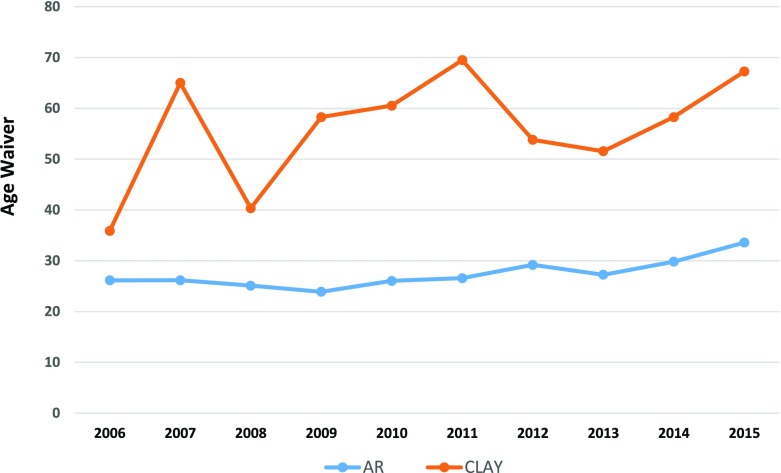
Arkansas Age Waivers (Age 14 and 15 yrs.) State vs. Clay County, 2006–2015

Age waivers issued in Arkansas for teens ages 14 and 15 years from 2006 to 2015 were analyzed, yielding an average state rate of 30.4 waivers per 1000 teens during this period (Arkansas Integrated Revenue Systems, 2016). Rates of age waiver issuance ranged by county from 11.9 to 56.0 per 1000 teens. Thirty one of seventy five counties in the state had issuance rates higher than the state average rate, with Clay County in northeast Arkansas issuing the most waivers (56 per 1000) (Fig. 1). The issuance of age waivers in Arkansas has slowly been increasing since 2006 (26.14 per 1000 in 2006 vs. 33.57 per 1000 in 2015) with a slightly higher increase annually since 2013. Over the entire period, the age waiver rate in Clay County increased fourfold while the overall state rate doubled (Fig. 2).

### Arkansas young teen driver fatality risks

Using FARS data, we assessed risk factors associated with fatal crashes among the twenty four, 14 and 15 year old, driver fatalities in Arkansas between 2004 and 2014. Due to the small overall number, no stable fatality rate could be generated. The number of age waivers is not included and the licensure status is not collected with fatal crash data. Most (79%) fatal crashes occurred among male drivers and 79% of the fatalities occurred between 7 and 9 AM and 3–6 PM, corresponding to peak transport times to and from school. Fifty-five percent of drivers who died in a fatal crash were unrestrained and of those, 46% were ejected from their vehicle. Alcohol involved fatalities accounted for 13% of 14 and 15 year old deaths (Table 1).

**Table 1:** Arkansas Fatality Risks, 2004–2014, 14 and 15 year old drivers, *N* = 24

	Male	Female
Age		
14 years old	29%	4%
15 years old	50%	21%
Seat Belt Use		
Yes	29%	4%
No	42%	13%
Missing	8%	4%
Ejection		
Yes	33%	13%
No	50%	8%
Time of Crash		
3:59 am-10:59 pm Allowable	58%	21%
11:pm-4 am Restricted	21%	0%
Alcohol Involved		
Yes	13%	0%
No	67%	21%

**Fig. 3:**
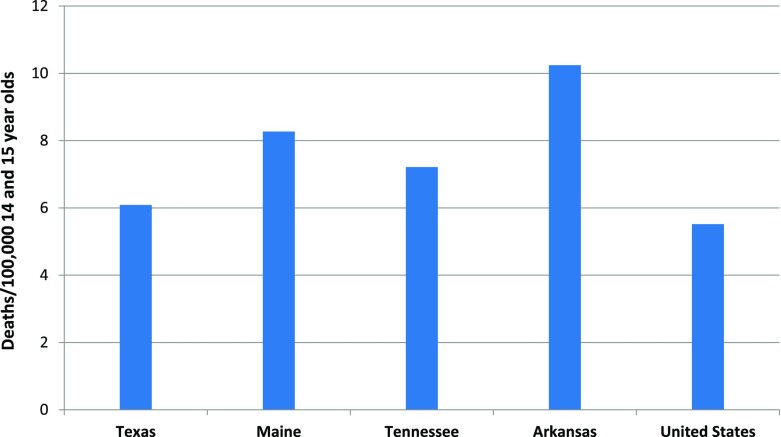
Motor Vehicle Traffic-related Death Rates, 14 and 15 year olds, By State, 2004–2014. Deaths/100,000 14 and 15 year olds

Texas, Maine, and Tennessee have similar age waiver policies in place for young drivers. Texas requires teen drivers to complete driver education and a parent is required to supervise the teens driving for at least 30 h of drive time (DMV, n.d.). Arkansas does not require teens to complete drivers’ education and teens are required to hold their permit for at least 6 months with no minimal supervised driving hours required. Between 2004 and 2014, all four states demonstrated higher motor vehicle death rates for teens ages 14 and 15 years old than the national rate of 5.52 per 100,000. Arkansas demonstrated the highest motor vehicle death rate (10.01 per 100,000) among these states and Texas had the lowest rate, 6 per 100,000 young teens, (Maine 8.7 per 100,000 young teens, Tennessee 7.21 per 100,000 young teens) (Centers for Disease Control and Prevention, 2009b) (Fig. 3). Seat belt use reported among the four states indicated Arkansas had the lowest seat belt use rate of 73% (Texas 98%, Maine 86%, Tennessee 86%).

## Discussion

Motor vehicle crashes continue to be the leading cause of death for teens’ ages 14 to 19 years old (Centers for Disease Control and Prevention, 2009a). While the Arkansas GDL has reduced fatal crashes for teens by 57.5% since passage in 2009 (Rouse et al., 2013), age waivers continue to pose a risk for 14 and 15 year old drivers. Age waivers prematurely graduate a 14 and 15 year old driver to an Intermediate Licensing stage of GDL where adult supervision is not required. Age waivers allow parents and young teens the opportunity to evade some of the restrictions under GDL and may make enforcement of GDL more difficult. Our study identified risks for these younger drivers and the need for targeted parent education regarding these risks.

In Arkansas, Driver Control Hearing Officers (DCHO) are required to issue age waivers if parents provide the correct necessary documentations. It is unclear whether many of the waivers are due to true need or convenience of the parents. The issuance of age waivers for drivers under 16 years old is both relatively common and increasing. Thirty one of seventy six Arkansas counties are above the average state rate of age waivers issued. There are also regional differences in the overall prevalence of the waivers, indicating possible differences in community norms or awareness of risks. Fatality risks for 14 and 15 year olds in Arkansas are higher among male drivers, and fatal crashes are most common during driving times that would typically be allowed under waivers. There is reason for concern about the effect of age waivers for 14–15 year olds given the high fatality rates in this age group in Arkansas.

Arkansas has a higher crash fatality rate than 3 other states, and some Arkansas counties have a high rate of age waivers issued. Contributing factors may include the state’s lack of a standardized or mandated driver education program, minimal supervision during requirement for licensure, overall lower seat belt use, and the presence of age waivers. Other states, such as Texas, require driver education and 30 h supervised driving prior to licensure (DMV, n.d.). Based on current data, we are unable to specifically calculate attributable risk of age waivers in the overall pattern of teen motor vehicle crash fatalities.

Little published information has been available about licensing for 14 and 15 year old drivers and associated risks. This may be due in part to the limited number of states allowing these waivers: only nine of 50 states currently allow a mechanism for 14 or 15 year olds to obtain a drivers’ license (Insurance Institute for Highway Safety, 2017). Based on our study, however, the presence of unsupervised, very young drivers on the road requires further attention. There are several opportunities for improved safety, including education of parents at the time of a waiver request to ensure they understand the risks posed by early licensure, as well as examination of policies allowing age waivers for younger drivers.

## Conclusion

This study is the first to define the extent of issued age waivers and review some of the associated risks for extremely young drivers. The results identified opportunities for both parental educational and policy changes. Further research is needed to identify strategies to reduce demand for and issuance of age waivers for 14 and 15 year olds.

## Abbreviations

DCHO: Driver Control Hearing Officer
FARS: Fatal Analysis Reporting System
GDL: Graduated Driver Licensing
IIHS: Insurance Institute Highway Safety
NHTSA: National Highway Traffic Safety Administration
